# Anatomic consideration of the radial nerve in relation to humeral length for unilateral external fixation: a retrospective study using magnetic resonance imaging findings in korean

**DOI:** 10.1186/s12891-023-06474-y

**Published:** 2023-05-15

**Authors:** Jae Jung Min, Young Jin Ryu, Ki Hyuk Sung, Jisoo Lee, Ji Young Kim, Moon Seok Park

**Affiliations:** 1 Department of Orthopaedic surgery, Seoul National University Bundang Hospital, Gyeonggi-do, Republic of Korea; 2 Department of Radiology, Seoul National University Bundang Hospital, Gyeonggi-do, Republic of Korea; 3 Department of Orthopaedic surgery, Seoul National University College of Medicine, Seoul, Republic of Korea

## Abstract

**Background:**

This study aimed to present a safe zone for distal pin insertion for external fixation using magnetic resonance imaging (MRI) images.

**Methods:**

All patients who took at least one upper arm MRI from June 2003 to July 2021 were searched via a clinical data warehouse. For measuring the humerus length, proximal and distal landmarks were set as the highest protruding point of the humeral head and lowermost margin of ossified bone of the lateral condyle, respectively. For children or adolescents with incomplete ossification, the uppermost and lowermost ossified margin of the ossification centers were set as proximal and distal landmarks respectively. The anterior exit point (AEP) was defined as the location of the radial nerve exiting the lateral intermuscular septum to the anterior humerus and distance between the distal margin of the humerus and AEP was measured. The proportions between the AEP and full humeral length were calculated.

**Results:**

A total of 132 patients were enrolled for final analysis. The mean humerus length was 29.4 cm (range 12.9–34.6 cm). The mean distance between the ossified lateral condyle and AEP was 6.6 cm (range 3.0–10.6 cm). The mean ratio of the anterior exit point and humeral length was 22.5% (range 15.1–30.8%). The minimum ratio was 15.1%.

**Conclusion:**

A percutaneous distal pin insertion for humeral lengthening with an external fixator may be safely done within 15% length of the distal humerus. If pin insertion is required more proximal than distal 15% of the humeral shaft, an open procedure or preoperative radiographic assessment is advised to prevent iatrogenic radial nerve injury.

## Introduction

Since its first use in the 1970s, bone lengthening procedures have rapidly evolved over time [1–4]. Although not as widely used as leg bone lengthening, lengthening of the upper extremities has gained popularity among patients with short upper limbs caused by various etiologies. Lengthening methods are diverse, ranging from the use of classic ring-shaped Ilizarov apparatus to a monofixator with or without intramedullary nail installation [3, 5, 6].

Bone lengthening of the humerus is performed for cosmetic and/or functional reasons [1, 2, 7]. For most procedures, the insertion of a distal pin is performed without the aid of any radiographic device, meaning the location of the radial nerve is unknown. Therefore, there is a risk of radial nerve injury while inserting the distal pin. Although rare, there have been case reports of radial nerve neuropathy and direct axonotmesis of the nerve caused by blind distal pin insertion [8–11].

Many attempts have been made to determine a safe margin for distal humeral pin insertion, most of which were experiments on cadavers. These studies were performed on adult upper extremities and the results were presented as absolute values [12–16] (Table 1). These results are beneficial for determining the safe margin in adults; however, they are not applicable for patients with short humeri. In such cases, guidelines presented as proportions are much more valuable as the anatomy of these patients deviates from the norm.

**Table 1:** Previous studies performed to determine a safe margin for distal humeral pin insertion

Study	Year published	Study mode	Study modality	No. patients	Avg. age (yr)	Radial n. measurement	Result	Mean humeral length (cm)	MIN/MAX	Location of the radial nerve from the elbow joint (cm)	Limitations
Current study		Retrospective study	U/E MRI	136		AEP/humeral length	Proportion			15%	
Gerwin et al.	1996	Anatomical study	Cadaver	10		1. Radial n. at posterior humerus/humeral length 2. Distance of the radial n. piercing the intermuscular septum to the lateral condyle/humeral length	Absolute Proportion	28 ± 1.9		1. 14.2 ± 0.6 (51%) 2. 10.2 ± 0.4 (36%)	Adults
Kamineni et al.	2009	Anatomical study	Cadaver	70	67 (52–85)	Lateral radial n. height : distance b/w the most prominent point of the lateral epicondyle and the point where the radial n. crosses the humerus in the mid lateral plane	Absolute	TED 6.2 ± 0.6		10.2 ± 0.1 (7.5–12.9)	Adults
Sukegawa et al.	2017	Anatomical study	Cadaver	20	84 (66–97)	Distance from the lateral COR to the point where the nerve crosses the lateral border according to elbow flexion from 0°~130º	Absolute	30.1 (2.7–3.5)		0°; 9.4 ± 0.8 10°; 9.3 ± 0.7 50°; 9.3 ± 0.8 90°; 9.3 ± 0.8 130°; 9.4 ± 0.7	Adults
Ye et al.	2019	Anatomical study	Cadaver, CT	28	81	Distance from the elbow COR at a deviation angle of posterior 30°~anterior 45°	Absolute			8.7 ± 0.85 (6.8 ~ 9.9)	Adults
Bloom et al.	2014	Retrospective study	Elbow MRI, XR	22 (23 MRIs)	9 ± 4 (3–12)	Most proximally detected radial nerve on coronal MR / TED	Proportion			60% of TED	Children
O’Shea et al.	2019	Retrospective study	U/E MRI	53	1–17	Distance between the radial n. and osseous landmarks/ humeral length	Proportion			44.98 ± 3.68% of arm length	Children
Patra et al.	2021	Anatomical study	Cadaver	20 (40 limbs)	45–60	Point where the radial n. enters the anterior compartment in relation to the apices of triceps aponeurosis	Absolute			1.98 ± 0.6 (1.00-2.50)	Adults

There have been recent retrospective studies using noninvasive methods to evaluate the course of the radial nerve. Many imaging techniques exist, and MRI is considered as a reliable and valid means of visualizing the radial nerve [17]. Furthermore, studies using MRI have yielded reliable results [18, 19]. On MRI, the course of the radial nerve can be traced as it traverses along the posterior arm through the triceps muscles, around the lateral humeral cortex, then through the intermuscular septum between the brachioradialis and brachialis before it branches into the posterior interosseous nerve before anteriorly drifting away from the cortex [20].

Information on the location of the radial nerve in relation to the prominent confirmatory anatomic landmarks of the upper extremities, such as the elbow joint line, would be extremely advantageous for performing bone lengthening procedures. Therefore, the aim of this study **was** to map the location of the radial nerve in the distal part of the humerus using MRI to define a safe zone for distal pin insertion during external fixation of the upper extremities.

## Materials and methods

This retrospective study was approved by the Institutional Review Board of the Seoul National University Bundang Hospital (IRB No. B-2111-719-102) and adheres to the ethical principles of the Declaration of Helsinki. The need for informed consent was waived due to the retrospective nature of the study.

### Patient selection

**Fig. 1:**
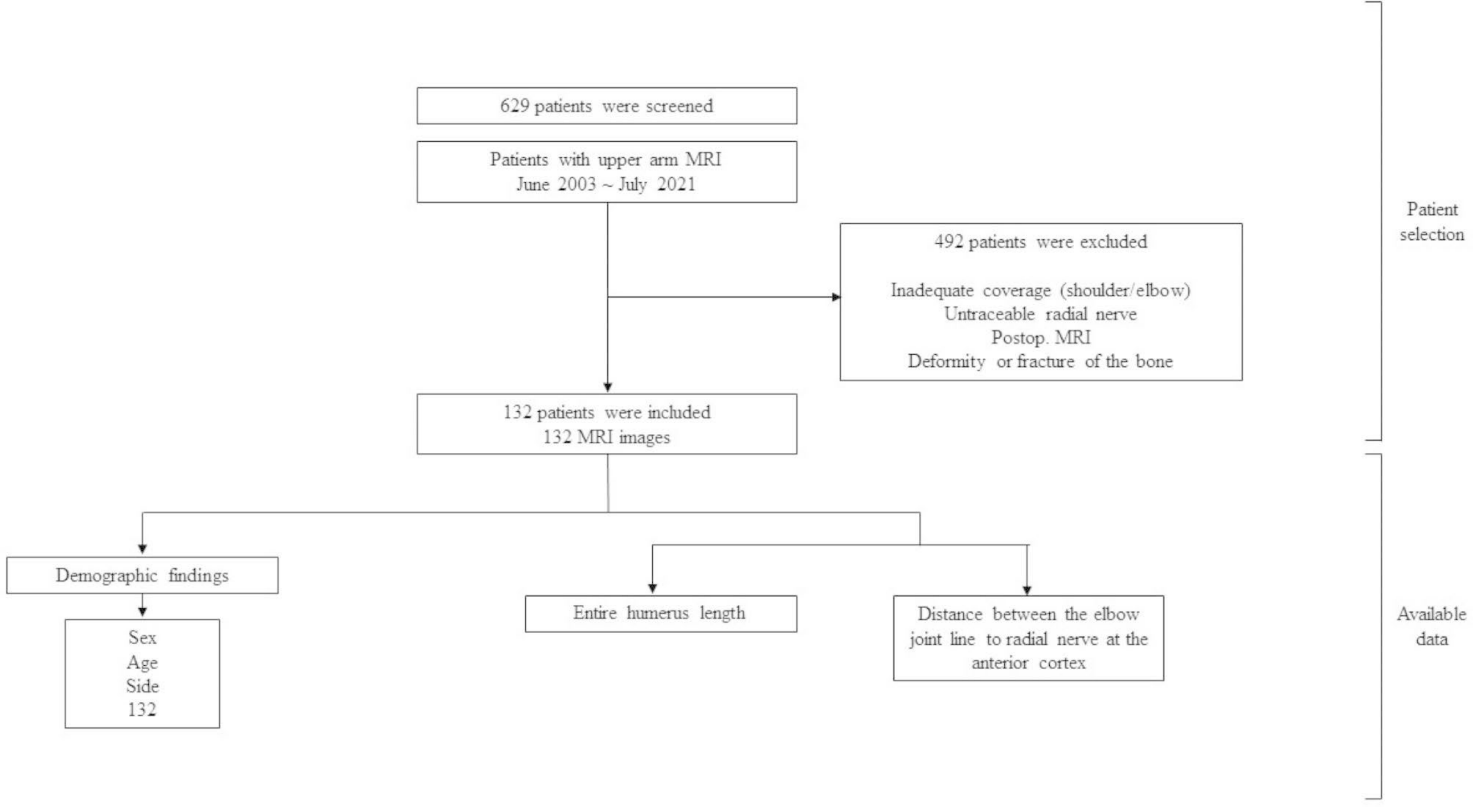
Flow diagram of patient selection

All patients who underwent at least one upper arm MRI from June 2003 to July 2021 were searched for via the clinical data warehouse of our hospital (Healthcare Information and Management Systems Society [HIMSS], stage 7). All MRI images were screened and images with the following were excluded: (1) inadequate coverage of either the proximal or distal joint, (2) untraceable radial nerve due to surrounding soft tissue pathology or inadequate axial coverage of the MRI, (3) images with fracture or deformity of the humerus, and (4) postoperative MRI images (Fig. 1).

### Imaging technique

The MRI examinations were performed on a 3.0-T (Ingenia, Ingenia CX, or Achieva; Philips Healthcare, Best, The Netherlands) or 1.5-T unit (Integra; Philips Healthcare, Best, The Netherlands) with extremity coils. In the MRI scanner, the patients were in the supine position with the arm supinated, the shoulder abducted and externally rotated. Axial and coronal T1-weighted images were used for radiologic measurement. The MR sequence parameters varied depending on the anatomical location of the lesion or clinical indication (axial T1-weighted image, section thickness, 3 to 7, intersection gap, 0.3 to 1; coronal T1-weighted image, section thickness, 3 to 4, intersection gap, 0.3 to 1).

### Radiographic measurements

**Fig. 2:**
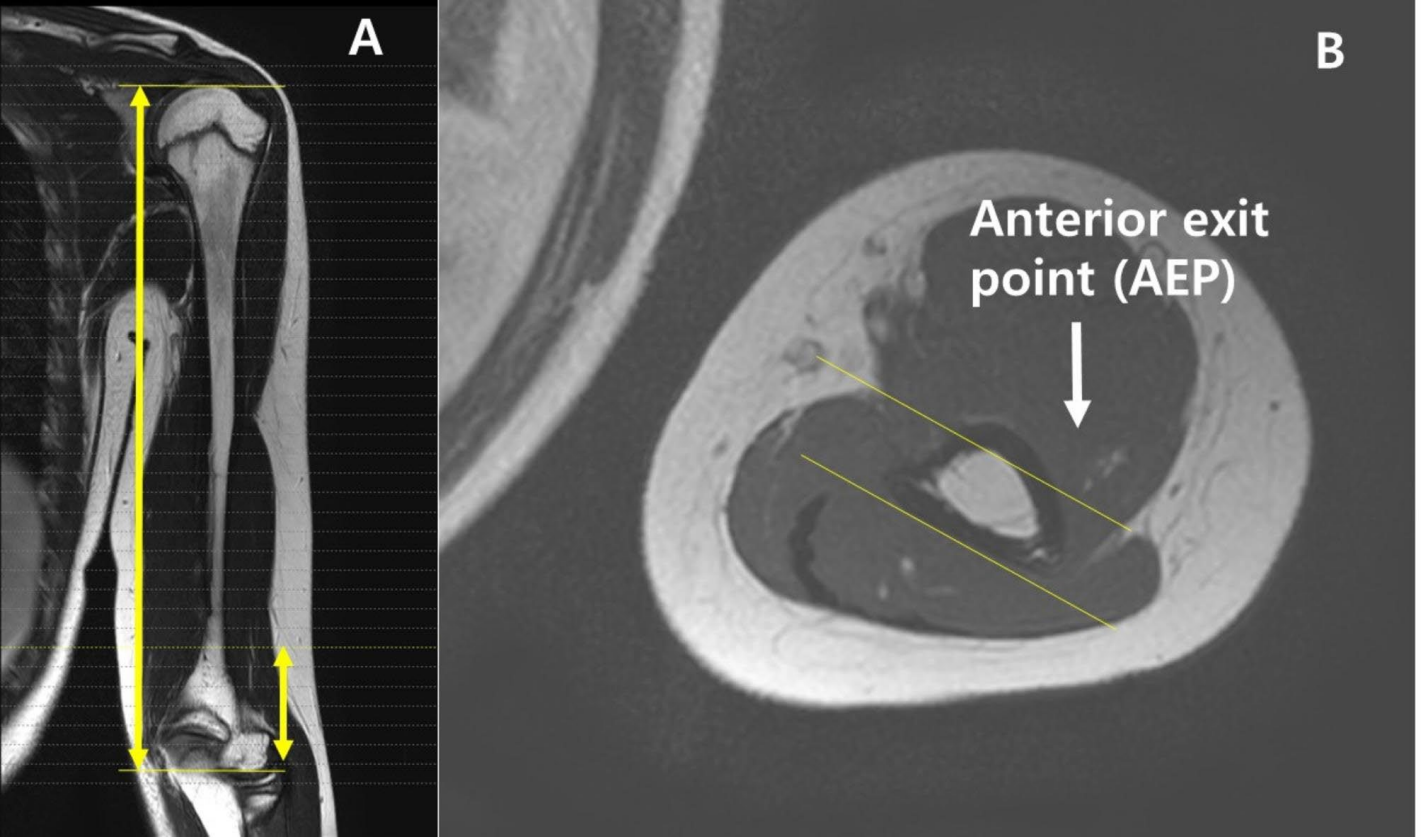
Defining landmarks for radiographic measurement. (a) The proximal landmark for measuring humerus length was set as the highest protruding point of the humeral head. The distal landmark for measuring humerus length was set as the lowermost margin of ossified bone of the lateral condyle. For children or adolescents with incomplete ossification, the landmark for measuring was set as the lowermost ossified margin of the ossification center of the capitellum. (b) The point where the nerve leaves the lateral intermuscular septum into the anterior humerus was designated as the anterior exit point (AE)

Before measuring, all authors held a consensus building session. The landmarks for measuring humerus length were set with consideration of the clinical setting in which plain radiographs were used rather than MRI scans. The proximal landmark for measuring humerus length was set as the highest protruding point of the humeral head. The distal landmark was set as the lowermost margin of ossified bone of the lateral condyle. For children or adolescents with incomplete ossification, the uppermost ossified margin of the ossification center of the humerus head was set as the proximal landmark and the lowermost ossified margin of the ossification center of the capitellum was set as the distal landmark (Fig. 2a). Then, MRI was cross-linked, and the radial nerves were traced from the proximal landmarks to the distal landmarks. The point at which the radial nerve leaves the lateral intermuscular septum into the anterior humerus was defined as the anterior exit point (AEP). The distance between the distal margin of the humerus and the AEP was measured (Fig. 2b).

After the consensus building session, two radiologists independently measured humerus length and the distances from the distal margin of the humerus to the AEP. All MRIs were evaluated on a picture archiving and communication system workstation (INFINITT Healthcare, Seoul, Korea).

### Statistical analysis

Descriptive statistics were used to summarize patient demographics and radiographic measurements. This included the mean of each patient’s humeral length and distance from the distal landmark to the AEP. The proportion of the AEP to the entire humerus length (AEP/HL) was calculated, and the average, minimum, and maximum values were obtained.

Reliability was assessed using intraclass correlations (ICCs), assuming single measurements and absolute agreement [21, 22]. Interobserver correlations were defined as follows: 0 to 0.24 was absent to poor, 0.25 to 0.49 was low, 0.50 to 0.69 was moderate, 0.7 to 0.89 was good, and 0.90 to 1.00 was excellent.

Final results were analyzed using the means of data measured by two observers, and data were presented as both absolute values and percentage proportions. Statistical analysis was performed using SPSS software (IBM Corp. Released 2019. IBM SPSS Statistics for Windows, Version 26.0 Armonk, NY: IBM Corp).

## Results

Overall, 629 patients were screened, and a total of 132 patients were enrolled for analysis (Fig. 1). The patient demographic characteristics are shown in Table 2, including the mean length of the humeri, mean distance between the ossified lateral condyle to the AEP and the mean ratio of the AEP and humeral length. **All selected patients were Korean.**

The interobserver reliability of MRI measurements of the AEP ranged from 0.873 to 0.934, with ICCs of 0.908 (Table 3).

**Table 2:** Demographic characteristics of the study population

	n = 132
Sex (Female/male)	72/59
Age (year)	47.8 ± 19.6 (range 1.8–84.8)
Bilaterality (Right/Left)	69/63
Humeral length (cm)	29.4 (range 12.9–34.6)
AEP (cm)	6.6 (range 3.0-10.6)
AEP/Humeral length (%)	22.5 (range 15.1–30.8)

AEP, anterior exit point

**Table 3:** Interobserver reliability of the radiographic measurements

	Interobserver reliability	
Measurement	ICC	95% CI
Humeral length	0.998	0.997–0.999
AEP	0.908	0.873–0.934
AEP/Humeral length	0.872	0.825–0.907

ICC, intraclass correlation coefficients; CI, confidence interval; AEP, anterior exit point

A subgroup analysis of patients according to age was performed using the same measurements which can be found in Table 4.

**Table 4:** Subgroup Analysis according to patient age

Variables	Values
Age < 18 years	18 patients
Age (year)	12.7 ± 5.5 (range 1.8–18.7)
Humeral length (cm)	26.8 (range 12.9–33.3)
AEP (cm)	6.1 (range 3.1–9.4)
AEP/humeral length (%)	22.7 (range 15.1–30.6)
Age > 18 years	114 patients
Age (year)	53.4 ± 14.6 (range 19.5–84.8)
Humeral length (cm)	29.9 (range 25.6–34.6)
AEP (cm)	6.7 (47.4-101.2)
AEP/humeral length (%)	22.4 (17.0-32.2)

AEP, anterior exit point

## Discussion

In our study of 132 patients, the minimum humeral length ranged from 12.9 to 25.6 cm. The minimum ratio between the AEP and entire humeral length ranged from 15.1 to 17.0%. This indicates that 15% of the distal lateral cortex of the humerus is void of any contact with the radial nerve and thus, percutaneous pin insertion can be performed safely. We chose to measure the distance between the distal landmark and the AEP because the nerve has already lost contact with the lateral humeral cortex at this location. Our reasoning was that by choosing a point where the nerve drifts away from the bony cortex, more accurate information of a “safe zone” would be provided. If a distal pin must be inserted proximally above the 15% range, an open procedure or preoperative radiographic assessment (using either MRI or ultrasonography [23]) is recommended to assess the course of radial nerve.

Several studies have been conducted in the hopes of defining a “safe zone” for distal pin insertion for external fixation of the upper arm [12–14]. However, most of these contained small study populations or were cadaveric studies done on adult populations [12–15] (Table 1), hindering the applicability of the results for use in younger patients. Additionally, results are usually given as an absolute distance from the elbow joint. Considering that most patients that require humeral lengthening have short humeri compared to the general population, values given in absolute distance lose clinical importance as the anatomy of these patients is deviated from the norm.

Few attempts have been made to assess the radial nerve in pediatric patients using MRI. Bloom et al. [18] evaluated the course of the radial nerve in relation to transepicondylar distance (TED) and noted the location of the radial nerve in terms of the angle between the nerve and the transepicondylar axis. The authors also tried to determine the relation between the lateral supracondylar ridge (LSR) and TED and concluded that the LSR is approximately 50% of the TED on average. Another retrospective assessment by O’Shea et al. [19] involved upper arm MRI analysis of 53 skeletally immature patients. They found that the radial nerve crossed the lateral humeral cortex at a distance equaling 44.98 ± 3.68% of humeral length and crossed from the posterior to the anterior compartment at approximately 35.27 ± 3.38% of humeral length. While these studies enlighten radial nerve anatomy in skeletally immature patients, they also highlight the danger zones for the radial nerve. Although the location of the radial nerve in approximation to the cortex has been defined, the location where the nerve drifts away from the bony cortex is not well established but essential for successful percutaneous pin fixation. Additionally, we deemed that the lower most value of the AEP/humeral length is more important than the average, as it is crucial to recognize the anatomic deviation of the location of the nerves so that percutaneous pin insertion can be performed safely.

The limitations of this study must be addressed. First, the study was retrospective and conducted in a single institution. Therefore, other imaging techniques, such as ultrasonography, that are also excellent in visualizing the radial nerve with far less cost could not be utilized. However, an assessment using MRI in such a large number of subjects, including subgroup analysis based on age, still provides valuable information on radial nerve anatomy. Second, the images analyzed did not include those of skeletal dysplasia or growth plate injuries. The proportions yielded in this analysis may differ in a population with skeletal dysplasia and/or growth arrest of either the upper or lower humerus. A future study involving analysis of the radial nerve in skeletal dysplasia or growth arrest injuries is necessary to evaluate the safe margins for this population subgroup and whether the safe margins for pin insertion adheres to the values obtained in this analysis. Third, muscle diseases such as sarcopenia was not excluded in the study as long as the tracking of radial nerve was possible. Although the result was not significantly affected given the method of this study, another analysis with exclusion of muscle disease may be done for further study.

## Conclusion

A percutaneous distal pin insertion for humeral lengthening with an external fixator may be safely done within 15% of the length of the distal humerus. If pin insertion is required proximal to 15% of the length of the humerus, an open procedure or preoperative radiographic assessment is recommended to prevent iatrogenic radial nerve injury.

## Data Availability

The datasets used and/or analysed during the current study are available from the corresponding author on reasonable request.

## Abbreviations

AEP: Anterior exit point
LSR: Lateral supracondylar ridge
MRI: Magnetic resonance imaging
TED: Transepicondylar distance
