# Correction: Anatomic consideration of the radial nerve in relation to humeral length for unilateral external fixation: a retrospective study using magnetic resonance imaging findings in korean

**DOI:** 10.1186/s12891-023-06560-1

**Published:** 2023-06-01

**Authors:** Jae Jung Min, Young Jin Ryu, Ki Hyuk Sung, Jisoo Lee, Ji Young Kim, Moon Seok Park

**Affiliations:** 1 Department of Orthopaedic surgery, Seoul National University Bundang Hospital, Gyeonggi-do, Republic of Korea; 2 Department of Radiology, Seoul National University Bundang Hospital, Gyeonggi-do, Republic of Korea; 3 Department of Orthopaedic surgery, Seoul National University College of Medicine, Seoul, Republic of Korea

**Correction:** ***BMC Musculoskelet Disord*** **24, 380 (2023)**

10.1186/s12891-023-06474-y

Following publication of the original article [1], the authors would like to make the following changes to affiliations:

1. Affiliation 2 should be changed to “*Department of Radiology, Seoul National University Bundang Hospital, Gyeonggi-do, Republic of Korea*”
2. Additional affiliation to Dr. Moon Seok Park (*Department of Orthopaedic surgery, Seoul National University College of Medicine, Seoul, Republic of Korea*)

The affiliations have been updated above and the original article [1] has been corrected.
